# Seroprevalence and Molecular Identification of *Brucella* spp. in Bovines in Pakistan—Investigating Association With Risk Factors Using Machine Learning

**DOI:** 10.3389/fvets.2020.594498

**Published:** 2020-12-02

**Authors:** Aman Ullah Khan, Falk Melzer, Ashraf Hendam, Ashraf E. Sayour, Iahtasham Khan, Mandy C. Elschner, Muhammad Younus, Syed Ehtisham-ul-Haque, Usman Waheed, Muhammad Farooq, Shahzad Ali, Heinrich Neubauer, Hosny El-Adawy

**Affiliations:** ^1^Friedrich-Loeffler-Institut, Institute of Bacterial Infections and Zoonoses, Jena, Germany; ^2^Department of Pathobiology, College of Veterinary and Animal Sciences, Jhang, Pakistan; ^3^Climate Change Information Center, Renewable Energy and Expert Systems (CCICREES), Agricultural Research Center, Giza, Egypt; ^4^Department of Brucellosis, Animal Health Research Institute, Agricultural Research Center, Giza, Egypt; ^5^Section of Epidemiology and Public Health, University of Veterinary and Animal Sciences, Lahore Sub-Campus, Jhang, Pakistan; ^6^Department of Pathobiology, KBCMA College of Veterinary and Animal Sciences, Narowal, Pakistan; ^7^Wildlife Epidemiology and Molecular Microbiology Laboratory (One Health Research Group), Discipline of Zoology Department of Wildlife & Ecology, University of Veterinary and Animal Sciences, Lahore, Pakistan; ^8^Faculty of Veterinary Medicine, Kafrelsheikh University, Kafr El-Sheikh, Egypt

**Keywords:** Brucellosis, *Brucella abortus*, bovines, seroprevalence, risk factors, machine learning

## Abstract

Bovine brucellosis is a global zoonosis of public health importance. It is an endemic disease in many developing countries including Pakistan. This study aimed to estimate the seroprevalence and molecular detection of bovine brucellosis and to assess the association of potential risk factors with test results. A total of 176 milk and 402 serum samples were collected from cattle and buffaloes in three districts of upper Punjab, Pakistan. Milk samples were investigated using milk ring test (MRT), while sera were tested by Rose–Bengal plate agglutination test (RBPT) and indirect enzyme-linked immunosorbent assay (i-ELISA). Real-time PCR was used for detection of *Brucella* DNA in investigated samples. Anti-*Brucella* antibodies were detected in 37 (21.02%) bovine milk samples using MRT and in 66 (16.4%) and 71 (17.7%) bovine sera using RBPT and i-ELISA, respectively. Real-time PCR detected *Brucella* DNA in 31 (7.71%) from a total of 402 bovine sera and identified as *Brucella abortus*. Seroprevalence and molecular identification of bovine brucellosis varied in some regions in Pakistan. With the use of machine learning, the association of test results with risk factors including age, animal species/type, herd size, history of abortion, pregnancy status, lactation status, and geographical location was analyzed. Machine learning confirmed a real observation that lactation status was found to be the highest significant factor, while abortion, age, and pregnancy came second in terms of significance. To the authors' best knowledge, this is the first time to use machine learning to assess brucellosis in Pakistan; this is a model that can be applied for other developing countries in the future. The development of control strategies for bovine brucellosis through the implementation of uninterrupted surveillance and interactive extension programs in Pakistan is highly recommended.

## Introduction

Brucellosis is a global widespread zoonotic disease caused by the Gram-negative, facultative intracellular bacterium *Brucella* (1, 2). In animals, the disease is characterized by full-term abortion, infertility, mastitis, and decreased milk production in females and orchitis/epididymitis in males (3, 4). However, the infection may stay asymptomatic, and the infected animals may remain undiagnosed (1). Brucellosis is usually transmitted in animals either by contact or through ingestion of contaminated feed and water while in humans by either direct contact with infected animals or ingestion of contaminated animal products (1, 5–8).

The *Brucella* genus includes 12 recognized species with varying host preferences, pathogenicity, and epidemiology (4, 9). Primarily, *Brucella abortus* causes infection in cattle and buffaloes, *Brucella melitensis* in sheep and goats, and *Brucella suis* in pigs. However, cross-species infection between different animal species is also possible (10–12). Most infected animals spontaneously shed bacteria in urine, milk, and vaginal secretions. Brucellosis may lead to severe economic losses through abortion, or stillbirth, death of young stock, and extra costs for breeding improvements (12).

Confirmatory diagnosis depends on laboratory-based examination of clinical specimens, e.g., serum or milk. For serological testing usually, a screening test of high sensitivity is followed by a confirmatory test of high specificity (13). Apart from being time and proficiency demanding, the culture of *Brucella* is hazardous to laboratory personnel and requires a biosafety level-3 facility (14). Thus, the detection of *Brucella* DNA by PCR in clinical samples is preferred as a tool for definitive diagnosis of brucellosis (15).

Brucellosis is endemic in many countries in Africa, Middle East, Mediterranean Basin, Asia, and Latin America with high records in humans in the Middle East and central Asian regions (16). It is endemic in Pakistan where it affects various livestock species and humans (12, 17). Disease burden particularly in bovines is increasing due to commercialization, urbanization, and increasing trends of extensive livestock farming (18, 19). Few studies on animal brucellosis are available relating particularly to the region of Northern Punjab. The overall seroprevalence of brucellosis in Pakistan was 3.25–4.4% in livestock (12). With the use of different serological tests, the seroprevalence in cattle and buffaloes was 4.4–5.06 and 5.2–5.6%, respectively (1, 12, 20). However, some studies reported seroprevalences of up to 31.9% in cattle and up to 47.19% in buffaloes particularly in those animals kept at organized dairy farms (21, 22).

Bearing in mind the economic burden of bovine brucellosis, the present study was designed to determine the seroprevalence, prevailing *Brucella* spp., and to study the relation between risk factors and the results of diagnostic tests in three districts of Punjab, Pakistan, by using statistical analysis and machine learning.

## Materials and Methods

### Study Area

**Figure 1:**
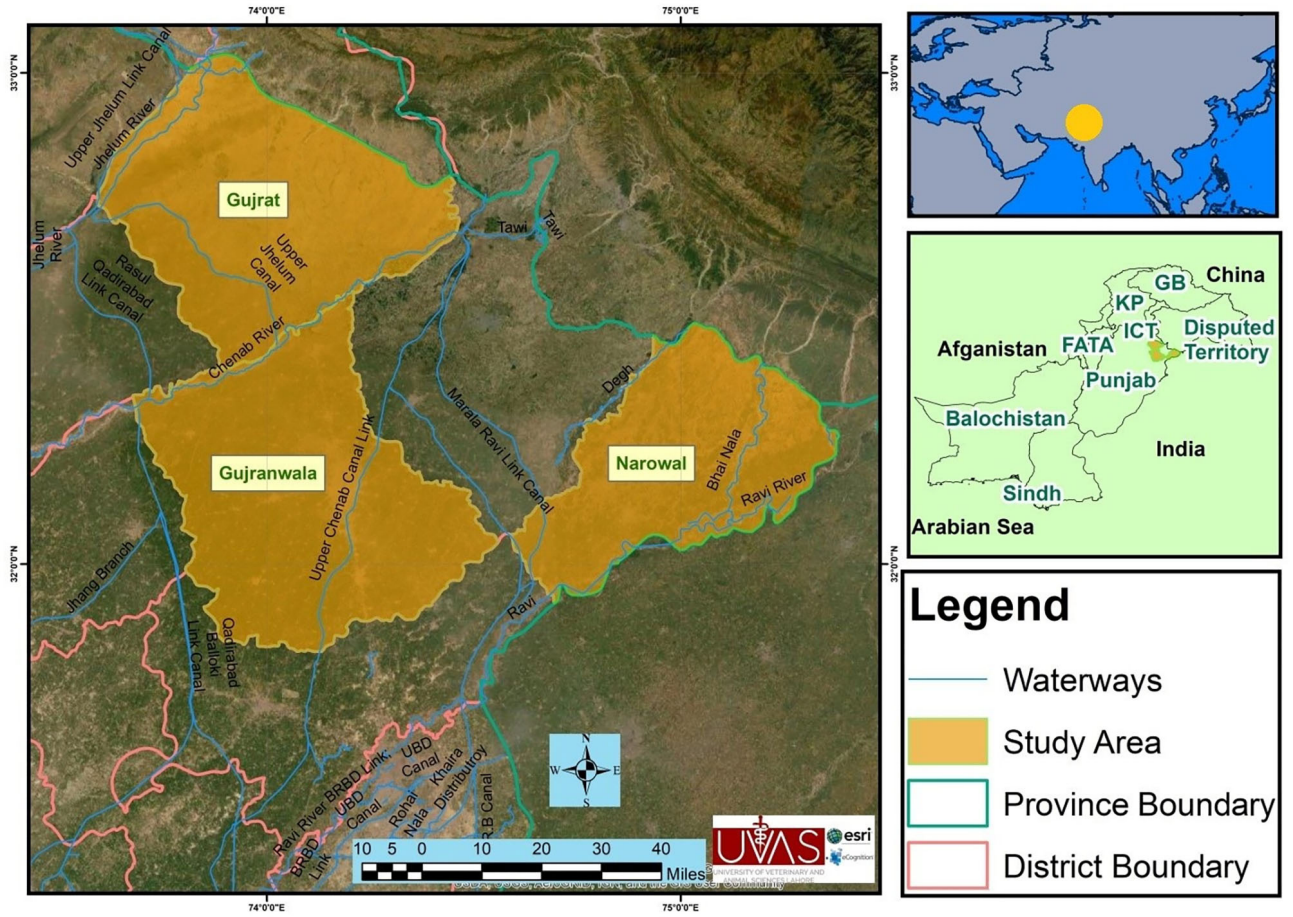
Map of the study area showing districts Gujranwala, Gujrat, and Narowal of upper Punjab, Pakistan.

The study was conducted in three selected districts of upper Punjab (Narowal, Gujranwala, and Gujrat), Pakistan (Figure 1). These three districts have high numbers of animals without previous report on brucellosis. The majority of the rural livelihood relies on small holding dairies averaging 10–15 animals. The Narowal District (32.2730°N, 75.0611°E) is located northwest to the Sialkot District and bordered at the north by Jammu and Kashmir India and at the southeast by the river Ravi and the Gurdaspur District of India. According to the 9211 Virtual Governance System, the livestock of this district include cattle (163,900), buffaloes (287,133), sheep (43,156), goats (122,886), and equines (31,956).

Gujranwala (32.1566°N, 74.1240E) is located north of the nearby provincial capital of Lahore. The city is Pakistan's fifth most populous metropolitan area. According to the 9211 Virtual Governance System, the livestock of this district include cattle (196,148), buffaloes (574,962), sheep (93,095), goats (97,636), equines (14,992), and camels (78 only).

Gujrat (32.5731°N, 74.1005°E) is situated at the shore of the river Chenab along the nearby cities of Sialkot and Gujranwala and forms part of the so-called “Golden Triangle” of industrial cities with export-oriented economies. According to the 9211 Virtual Governance System, the livestock of this district includes cattle (155,346), buffaloes (304,682), sheep (25,388), goats (125,887), equines (40,080), and camels (62 only).

Important livestock breeds of these districts are Sahiwal and cross-bred cattle, Nili Ravi buffaloes, Teddy and Beetal goats, and Lohi and Kajli sheep. The prevalent diseases of these regions related to livestock are milk fever, mastitis, Degnala disease (aspergillosis), bovine ephemeral fever, contagious caprine pleuropneumonia (CCPP), peste des petits ruminants (PPR), hemorrhagic septicemia (HS), and foot and mouth disease (FMD). Vaccination to these diseases (CCPP, PPR, HS, and FMD) is applied (23). The land of these districts is irrigated by the rivers Ravi and Chenab (https://irrigation.punjab.gov.pk/).

### Ethical Approval

This study was approved by the ethical committee of the College of Veterinary & Animal Sciences, Jhang University of Veterinary & Animal Sciences, Lahore, Pakistan via approval number CS/481. Oral and written consent was also taken from each farmer before blood and milk sample collection.

### Blood Samples

In this study, 402 blood samples were collected from cattle (*Bos taurus, n* = 208) and domestic water buffaloes (*Bubalus bubalis, n* = 194) located in the districts of Narowal, Gujranwala, and Gujrat in the period March to October 2015. Approximately 4–5 ml of blood were collected aseptically from the jugular vein of each animal in a blood vacutainer containing gel with clot-activating factors (Bio-One®, China) that were placed at room temperature for more than 0.5 h and stored immediately at 4°C in an icebox and were transported to the Epidemiology and Public Health Laboratory, College of Veterinary and Animal Sciences, Jhang, Pakistan. All tubes were centrifuged (Eppendorf, Germany) at 5,000 rpm for 5 min for serum separation. After centrifugation, the supernatants were collected in sterile Eppendorf tubes (1.5 ml) by pipettes and stored at −20°C for further analysis.

### Milk Samples

One hundred seventy-six milk samples were collected from the above-mentioned animals including 107 lactating cattle and 69 buffaloes located in the districts of Narowal, Gujranwala, and Gujrat. Samples were collected in the period March to October 2015. These milk samples were collected from animals that were in lactation with apparently healthy non-mastitic milk and from which serum samples were also collected. Five milliliters of milk was collected aseptically with a 10-ml syringe, preserved in an icebox, and transported to the Epidemiology and Public Health Laboratory, College of Veterinary and Animal Sciences, Jhang. Samples were kept at 4°C for 24 h prior to the investigation.

### Epidemiological Data Collection

The demographic (districts) and descriptive epidemiological data related to risk factors (i.e., species, gender, age, pregnancy status, lactation status, herd size, history of abortion, reproductive problems, vaccination history, and health conditions) were collected using a pre-structured questionnaire (Supplementary Material 1).

### Analysis of Milk Samples

Milk samples were investigated by milk ring test (MRT). The MRT antigen was procured from the Veterinary Research Institute Lahore, Pakistan. As per the manufacturer's recommendations, the MRT antigen was kept at room temperature 1 h before use. The MRT antigen was standardized against the OIE International Standard Serum (OIEISS) to give a positive result at an OIEISS dilution of 1:500 and a negative reaction at 1:1,000. One milliliter of milk sample was added to a test tube, and then 30 μl of antigen was added, mixed, and incubated at 37°C for 1 h. A sample having a change in color at the top of milk was considered positive (4).

### Analysis of Serum Samples

All sera were screened for anti-*Brucella* antibodies by Rose–Bengal agglutination test (RBPT) (RBPT antigen, Veterinary Research Institute, Lahore, Pakistan) and i-ELISA (IDEXX Brucellosis Serum Ab Test kit, IDEXX Laboratories, Inc. Westbrook, United States) according to the manufacturer's recommendations. The RBPT antigen was standardized against the OIEISS to give a positive reaction at a dilution of 1:45 and a negative reaction at a dilution of 1:55. The i-ELISA was calibrated according to the OIE specifications to correctly detect the OIE ELISA strong positive standard serum (OIEELISAspSS) (4). The OD value was taken using the ELISA reader (xMark™ microplate absorbance spectrophotometer, BIO-RAD, USA) at 450-nm wavelength. With the help of OD values, the SP ratio was calculated from the formula:

$$\begin{array}{r} \text{SP}=\frac{\text{Mean OD sample x }100}{\text{Mean OD of positive control}} \end{array}$$

Serum samples with SP < 25% were considered negative. Samples with SP ≥ 25% were regarded as positive.

### Molecular Detection of *Brucella* spp.

DNA was extracted from all serum samples using the QIAamp DNA Mini Kit (QIAGEN, Hilden, Germany) according to the instructions of the manufacturer.

Genus *Brucella* and species-specific (*Brucella abortus* and *Brucella melitensis*) real-time PCRs were used for the detection of *Brucella* DNA (24). PCR was performed using primer and probe (Jena Bioscience GmbH, Germany) sets as given in Table 1.

**Table 1:** Primers and probes sequences used in real-time PCR assays for the detection of *Brucella* spp., *Brucella abortus*, and *Brucella melitensis* in bovine, Pakistan.

**Target**	**Primer and probe sequences**		**Reference**
*Brucella* spp.	**5′-**GCT CGG TTG CCA ATA TCA ATG C**-3′** **5′-**GGG TAA AGC GTC GCC AGA AG**-3′** 6-FAM-AAA TCT TCC ACC TTG CCC TTG CCA TCA-MGB	Forward Reverse Probe	(24)
*B. abortus*	**5′-**GCG GCT TTT CTA CGG TAT TC**-3′** **5′-**CAT GCG CTA TGA TCT GGT TAC G**-3′** Hex-CGC TCA TGC TCG CCA GAC TTC AAT G-BHQ1-3	Forward Reverse Probe	
*B. melitensis*	**5′-**AAC AAG CGG CAC CCC TAA AA**-3′** **5′-**CAT GCG CTA TGA TCT GGT TAC G**-3′** Cy5-CAG GAG TGT TTC GGC TCA GAA TAA TCC ACA-BHQ2-3′	Forward Reverse Probe	

The PCR protocol was modified (volume and temperature) than previously published to obtain the most optimal results where DNA used in this study as template was extracted from serum not from bacterial colonies. Briefly, PCR reaction was performed in 15 μl of multiplex PCR mixture with 2× TaqMan™ environmental master mix (Applied Biosystems®, Darmstadt, Germany), 0.2 μM of each primer, 0.1 μM of each probe, and 5 μl of template DNA. Amplification and real-time fluorescence detection were carried out on a StepOnePlus™ Real-Time PCR System (Applied Biosystems®, Germany). The reaction conditions were as follows: decontamination at 50°C for 2 min, initial denaturation at 95°C for 10 min followed by 50 cycles of denaturing at 95°C for 25 s, and annealing/elongation at 57°C (*B. abortus* and *B. melitensis*) (24) for 1 min. Sample data scores were confirmed by visual inspection of graphical plots and cycle threshold (CT) values for each sample. CT values below 38 were considered positive. Reference strains of *B. abortus* S-99 (ATCC 23448), *B. melitensis* 16M (ATCC 23456) and *Brucella suis* biovar 1 (ATCC 23444) were used as positive controls. Reference strains of *Escherichia coli* (ATCC 25922), *Staphylococcus aureus* (ATCC 25923), and *Ochrobactrum intermedium* (DSM 17986) were used as negative controls.

### Studying the Relationship Between Risk Factors and Diagnostic Test Results Using Statistical Analysis by Pearson's Chi-Squared Test

Correlation of potential risk factors (geographical location, animal species, age, herd size, and reproductive disease/problem) with molecular detection of *Brucella* DNA was analyzed using Pearson's chi-squared test (χ^2^). The estimation of χ^2^ was done using RStudio Version 1.1.463.

### Studying the Relationship Between Risk Factors and Diagnostic Test Results Using Machine Learning

Machine learning has been used widely in prediction, clustering, and classification based on features. One of the powerful machine learning techniques is decision tree (25), which is used for modeling and classifying data. Decision tree was particularly chosen as a suitable machine learning technique for classifying the current categorical small-sized data. It also builds a model for the features (risk factors) based on their significance. The feature set represents the dependent variables, while serum test results were used as the independent or target variable. Exploring the significance of the features recorded for animals under sampling is the objective of using machine learning in this work. Nine categorical features representing the dependent variables were recorded for the 402 animals in the current work. These features were location, genus, species, sex, age, herd size, reproductive disease/problem, history of abortion, pregnancy, and lactation.

Decision tree technique is not only a classifier but also a model that can be visually seen and easily understood, which is important to the inferences beyond the model. It also explains the rules used for dividing data using inferred rules for this division. The aim was to predict a value of certain variables based on a created model. It uses training samples consisting of features (attributes). The data were divided into subsets, where data in lower subsets were purer than data in upper subsets. The attribute chosen to be represented in a node was the attribute with the highest information gain (Gini) of all attributes. The resulted model was represented in a tree-like shape, where internal node was a test conducted on an attribute, branches were the results of the test, and leaf nodes represented the decision taken. A pipeline was proposed by Fountain-Jones et al. (26) for using machine learning on exposure data (26). This pipeline consists of steps to be applied on exposure data in order to discover significant patterns in it. The following steps demonstrate to conduct machine learning on recorded data.

#### Data Pre-processing

All features are categorical so they need to be encoded or converted to numeric values. This conversion is shown in Table 2.

**Table 2:** Conversion of risk factor categories into numeric values for machine learning model.

**Risk factor (attribute)**	**Category**	**Value**
Location	Gujranwala	1
	Gujrat	2
	Narowal	3
Genus/species	Cattle (*Bos taurus*)	1
	Buffalo (*Bos bubalus*)	2
Sex	Male	1
	Female	2
Age	7–10 years	1
	3–7 years	2
	2–3 years	3
Herd size	1–10	1
	11–30	2
	>30	3
Reproductive disease/problem	Yes	1
	No	2
History of abortion	Yes	1
	No	2
Pregnancy	Yes	1
	No	2
Lactation	Yes	1
	No	2

#### Model Training

**Figure 2:**
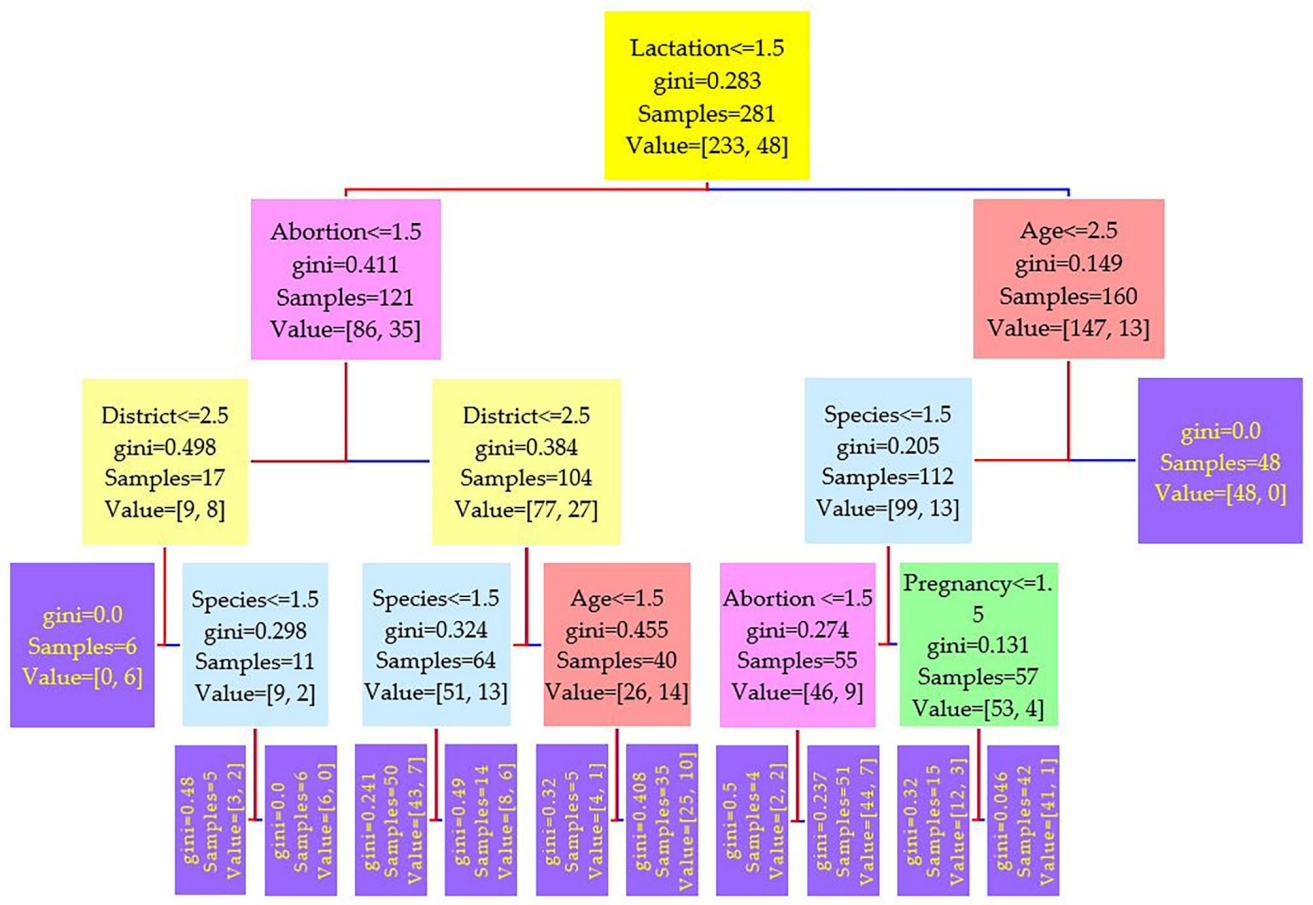
Decision tree for the Rose–Bengal plate agglutination test (RBPT) performed on 208 cattle (*Bos taurus*) and 194 buffaloes (*Bos bubalus*) from Narowal, Gujranwala, and Gujrat districts, Pakistan. The decision tree of RBPT reveals that the root node is lactation attribute and in the second level come attributes age and abortion.

**Figure 3:**
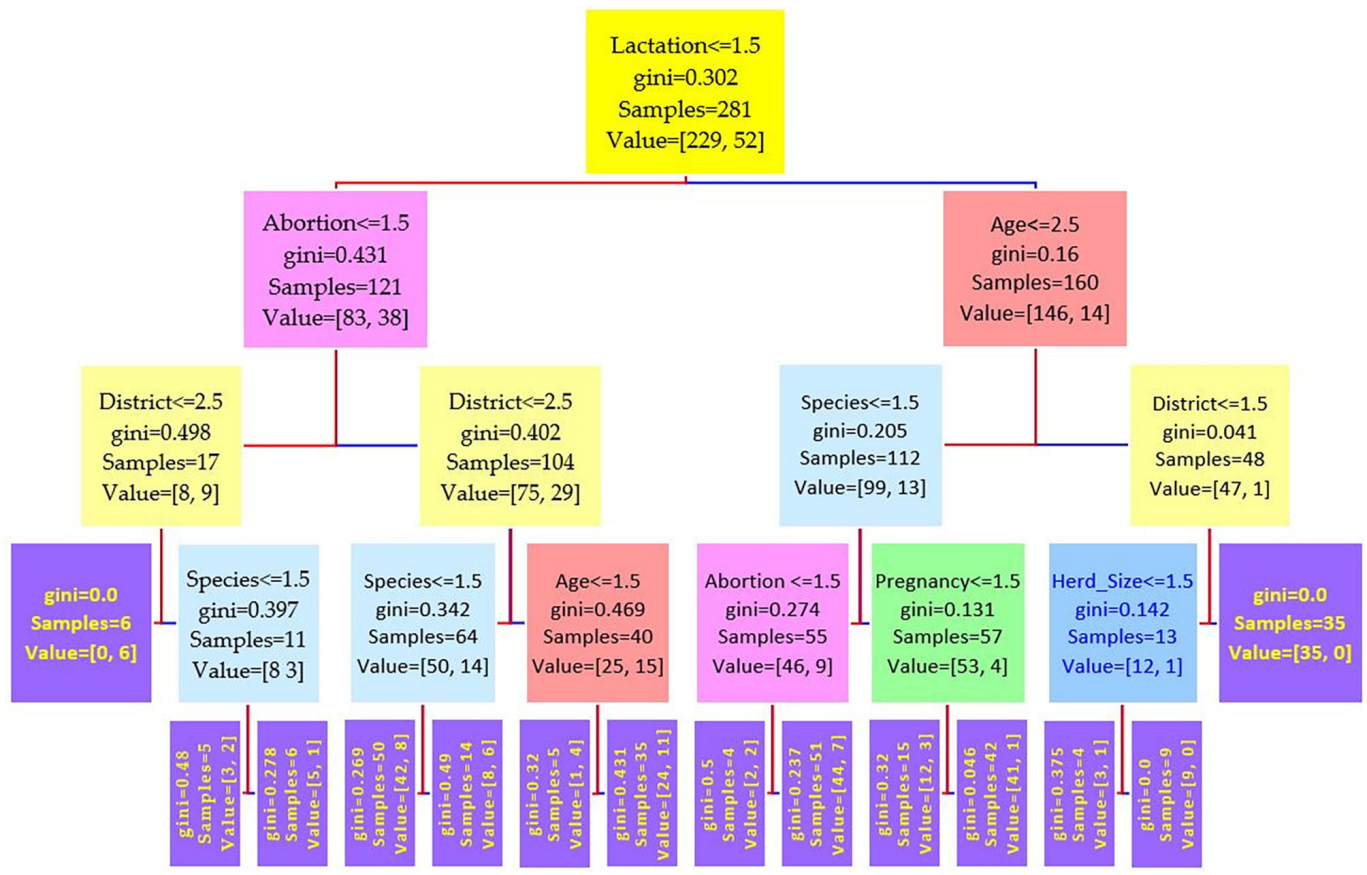
Decision tree for the i-ELISA performed on 208 cattle (*Bos taurus*) and 194 buffaloes (*Bos bubalus*) from Narowal, Gujranwala, and Gujrat districts, Pakistan. The decision tree of i-ELISA reveals that the root node is lactation attribute and in the second level come attributes age and abortion.

**Figure 4:**
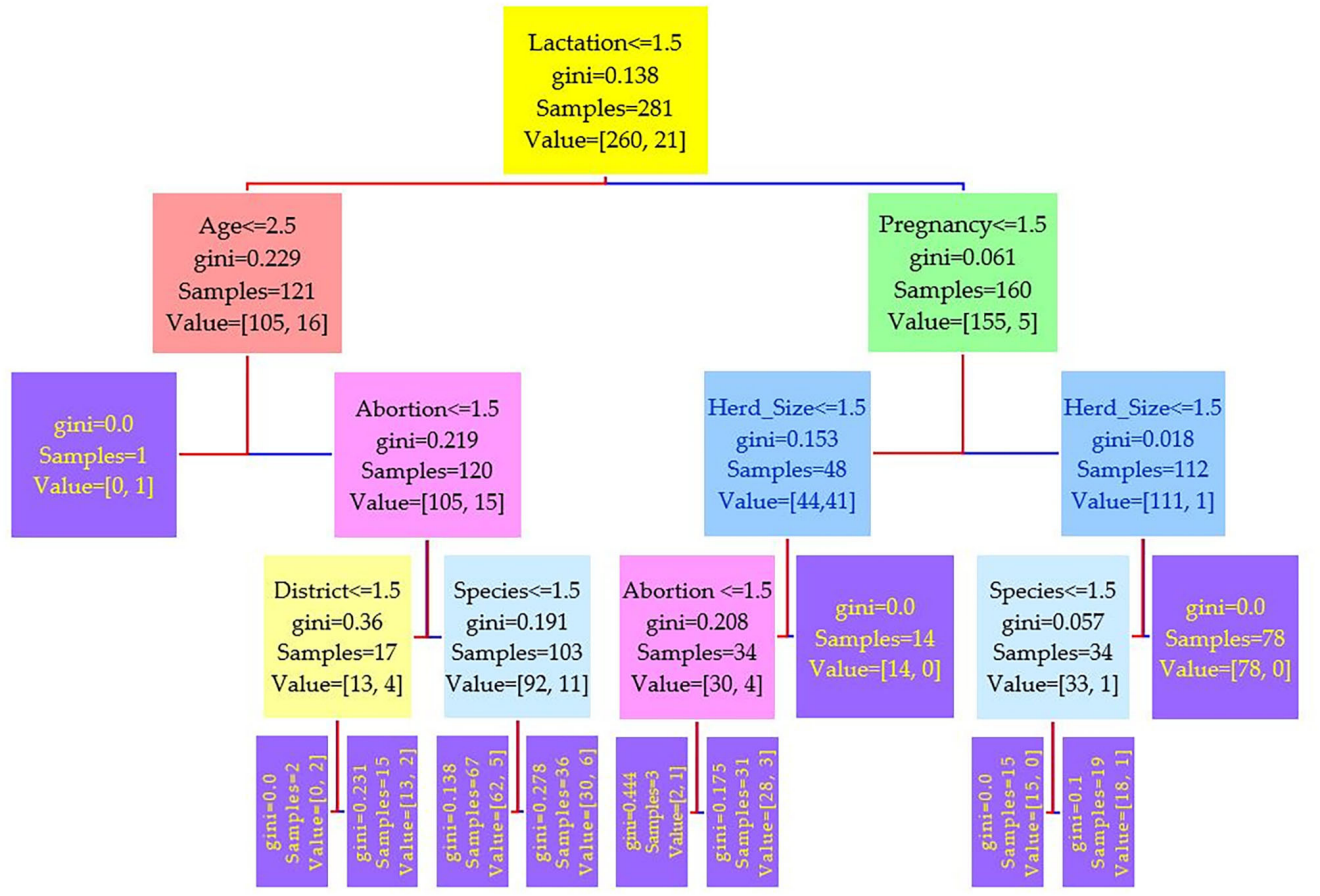
Decision tree for the real-time PCR performed on 208 cattle (*Bos taurus*) and 194 buffaloes (*Bos bubalus*) from Narowal, Gujranwala, and Gujrat districts, Pakistan. The decision tree of real-time PCR reveals that the root node is lactation attribute and in the second level come attributes age and pregnancy.

The data were divided into a training set of 281 cases (70%) and a test set of 121 cases (30%). Three different runs have been conducted on the features, and the results of three serum tests RBPT, i-ELISA, and real-time PCR were used as the independent variable. Three different trees resulted from the experiment and are shown in Figures 2–4, where the maximum depth of the trees was set to 4 in order to fit the presentation.

#### Model Performance Evaluation

Performance of the machine learning model was evaluated to find out if it actually worked and if it gave trustworthy predictions on new data other than the data it was trained on. The performance metrics precision or positive predicted value (PPV), recall or negative predicted value (NPV), accuracy, and area under the receiver operating characteristic curve (AUC ROC) were calculated from prediction scores for the serum tests RBPT, i-ELISA, and real-time PCR.

## Results

### Prevalence of Anti-*Brucella* Antibodies in Bovine Milk and Serum

Out of 176 milk samples collected from lactating bovines, 37 (21.02%) were positive. Anti-*Brucella* antibodies were detected in 19 (17.7%) and 18 (26.08%) of 107 from cattle and 69 from buffaloes milk samples, respectively (Table 3). Moreover, 16.4% (66 out of 402) and 17.7% (71 out of 402) bovine sera were found positive for anti-*Brucella* antibodies using RBPT and i-ELISA, respectively (Table 3). A higher seropositivity was found in cattle (18.75 and 19.71%) than in buffaloes (13.91 and 15.46%) using RBPT and i-ELISA, respectively (Table 3).

**Table 3:** Seroprevalence and molecular detection of *Brucella* DNA in bovine milk and sera collected from Narowal, Gujranwala, and Gujrat districts of upper Punjab, Pakistan.

**District**	**Animal**	**Number of**	**Number of**	**Serological examination**			**Molecular identification**		
		**milk samples**	**serum samples**						
				MRT^*^No.	RBPT^*^No.	i-ELISA^*^No.	**Real-time PCR**	***Brucella***	**Cq/Ct**
				**(%)**	**(%)**	**(%)**	**No. (%)**	**spp**.	values^*^
Narowal	^++^Cattle	39	65	9 (23.1)	16 (24.6)	16 (24.6)	4 (6.15)	*Brucella abortus*	32, 35, 36, 32
	^+++^Buffalo	42	69	8 (19.04)	9 (13.04)	12 (17.4)	7 (10.1)	*B. abortus*	36, 32, 30, 32, 31, 29, 30
***Subtotal***		***81***	***134***	***17 (20.9)***	***25 (18.6)***	***28 (20.9)***	***11 (8.21)***		
Gujranwala	Cattle	30	70	5 (16.6)	11 (15.7)	13 (18.5)	4 (5.71)	*B. abortus*	35, 32, 32, 35,
	Buffalo	9	64	4 (44.4)	8 (12.5)	8 (12.5)	6 (6.37)	*B. abortus*	30, 31, 31, 35, 29, 33
***Subtotal***		***39***	***134***	***9 (23.07)***	***19 (14.17)***	***21 (15.6)***	***10 (7.46)***		
Gujrat	Cattle	38	73	5 (13.1)	12 (16.4)	12 (16.4)	5 (6.84)	*B. abortus*	32, 34, 37, 34, 30
	Buffalo	18	61	6 (33.3)	10 (16.3)	10 (16.3)	5 (8.19)	*B. abortus*	27, 36, 32, 32, 35
***Subtotal***		***56***	***134***	***11 (19.6)***	***22 (16.4)***	***22 (16.4)***	***10 (7.46)***		
**Grand-total**		**176**	**402**	**37 (21.02)**	**66 (16.4)**	**71 (17.7)**	**31 (7.7)**		

* *MRT, milk ring test; RBPT, Rose–Bengal plate agglutination test; i-ELISA, indirect ELISA; Cq/Ct values (cycle quantification/cycle threshold values): this is the number of PCR cycles at which the sample's amplification curve intersects in the beginning of its exponential phase with the threshold line. The threshold line is the level of detection or the point at which a reaction reaches a fluorescent intensity above background levels. Cq indicates how many cycles it took to detect a real signal from every sample. Each sample has a reaction curve, which is a plot of the number of cycles vs. fluorescence intensity*.

++ *Bos taurus*.

+++ *Bos bubalus*.

*Italic values indicates Subtotal (%)*.

**Figure 5:**
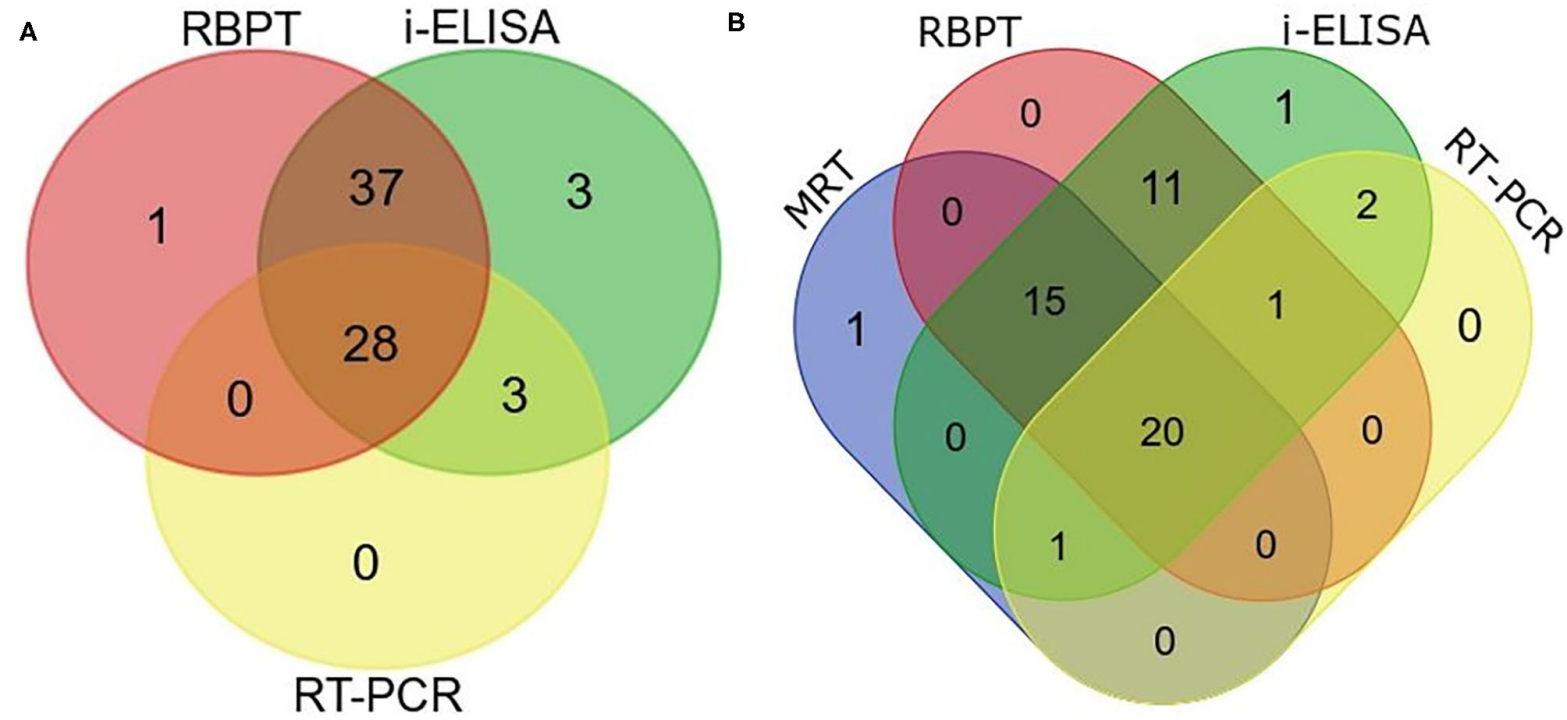
Venn diagram of correlations of serological tests and real-time PCR expressed as numbers of positive cattle (*Bos taurus*) and buffaloes (*Bos bubalus*) from the three target districts. **(A)** Results of all the 402 sera tested by Rose–Bengal plate agglutination test (RBPT), i-ELISA, and real-time PCR. **(B)** Results for the 176 lactating animals where milk ring test (MRT) was additionally performed.

Twenty eight animals were positive to all the three tests (Figure 5A). The real-time PCR was positive when both tests or either the RBPT or i-ELISA was positive. The RBPT and i-ELISA exclusively detected one and three animals, respectively. The i-ELISA detected the highest number of positive animals (71) followed by RBPT (66) and real-time PCR (31).

A total of 176 lactating animals were additionally tested by MRT. The test detected one animal exclusively and another animal that was only positive on the real-time PCR (Figure 5B). On the other hand, the MRT failed to detect 15 animals that were positive to one or more of the other tests.

### Detection of *Brucella* spp. DNA in Bovine Sera

*Brucella* DNA was detected in serum samples positive by RBPT or i-ELISA. *Brucella abortus* DNA was identified in 31 (7.7%) bovine sera (Table 3). *Brucella* DNA was not amplified from seronegative serum samples. A higher number of *Brucella* DNA positive samples were detected in 18 (9.27%) buffaloes than in 13 (6.25%) cattle. *Brucella* DNA was amplified from 8.21, 7.46, and 7.46% of bovine sera from Narowal, Gujranwala, and Gujrat districts, respectively.

### Statistical Results of Chi-Squared Test

The risk factors geographical location, animal species, age, herd size, and reproductive disease/problem revealed no statistical significance by Pearson's chi-squared test with diagnostic test results (RBPT, i-ELISA, and real-time PCR) as shown in Table 4.

**Table 4:** Statistical relationship of risk factors with detection of bovine brucellosis in Narowal, Gujranwala, and Gujrat districts of upper Punjab, Pakistan.

**Variable**	**Serological examination** ***n*** **(%)**		**Real-time-PCR**,
			***n* (%)**
	**RBPT**	**i-ELISA**	
**Geographical location**			
Gujranwala (*n* = 134)	19 (14.17)	21 (15.67)	10 (7.46)
Gujrat (*n* = 134)	22 (16.41)	22 (16.41)	10 (7.46)
Narowal (*n* = 134)	25 (18.65)	28 (20.89)	11 (8.21)
*p*-value^*^	0.9394	0.9276	
χ^2^	0.12495	0.15033	
Df	2		
95% CI	–	–	
OR	–	–	
**Species**			
Cattle (*n* = 208)	39 (18.75)	41 (19.71)	13 (6.25)
Buffaloes (*n* = 194)	27 (13.91)	30 (15.46)	18 (9.27)
*p*-value^*^	0.1141	0.1412	
χ^2^	2.4961	2.1652	
Df	1		
95% CI	0.7728451–5.2286033	0.7410196–4.8877704	
OR	1.985477	1.880346	
**Age**			
7–10 years (*n* = 98)	25 (25.51)	27 (27.55)	10 (10.2)
3–7 years (*n* = 236)	39 (16.52)	41 (17.37)	19 (8.05)
2–3 years (*n* = 68)	2 (2.94)	3 (4.41)	2 (2.94)
*p*-value^*^	0.6702	0.7936	
χ^2^	0.80047	0.4623	
Df	2		
95% CI	–	–	
OR	–	–	
**Herd size**			
1–10 (*n* = 128)	18 (14.06)	22 (17.18)	10 (7.81)
11–30 (*n* = 138)	22 (15.94)	22 (15.94)	9 (6.52)
>30 (*n* = 136)	26 (19.11)	27 (19.85)	12 (8.82)
*p*-value^*^	0.858	0.9798	
χ^2^	0.30622	0.040852	
Df	2		
95% CI	-	-	
OR	-	-	
**Reproductive disease/problem**			
Yes (*n* = 374)	66 (17.64)	70 (18.71)	31 (8.29)
No (28)	0	1 (3.57)	0
*p*-value^*^	-	0.5067	
χ^2^	-	0.44094	
Df	1		
95% CI	0–Inf	0.00000–89.19948	
OR	0	0	
**History of abortion**			
Yes (*n* = 39)	14 (35.89)	16 (41.02)	9 (23.07)
No (*n* = 363)	52 (14.32)	55 (15.15)	22 (6.06)
*p*-value^*^	0.3984	0.4829	
χ^2^	0.71309	0.49229	
Df	1		
95% CI	0.2257425–2.0039428	0.2505977–2.1199847	
OR	0.6610627	0.7135534	
**Pregnancy**			
Yes (*n* = 177)	37 (20.90)	39 (22.03)	16 (9.04)
No (*n* = 225)	29 (12.88)	32 (14.22)	15 (6.66)
*p*-value^*^	0.6816	0.7573	
χ^2^	0.16835	0.095536	
Df	1		
95% CI	0.464294–3.067330	0.4489188–2.8947825	
OR	1.1939	1.14108	
**Lactation**			
Yes (*n* = 176)	47 (26.70)	51 (28.97)	24 (13.63)
No (*n* = 226)	19 (8.41)	20 (8.85)	7 (3.097)
*p*-value^*^	0.5198	0.5563	
χ^2^	0.41423	0.34623	
Df	1		
95% CI	0.2247314–2.1213957	0.2336189–2.1616710	
OR	0.723859	0.745854	

* *Statistical value of significance: p ≤ 0.05*.

*χ^2^, Pearson's chi-squared test; Df, degree of freedom; CI, confidence interval; OR, odds ratio*.

### Performance Results of Machine Learning Model

The evaluation parameters of machine learning model revealed that the three tests recorded performance metrics very close to each other (Table 5). The PPVs were ≤50%, where the lowest was for real-time PCR (43%) while the highest was that of i-ELISA (50%) and RBPT was 44%. NPVs were also low and close to each other, where the highest value was 42% for i-ELISA while the lowest was 30% for real-time PCR, and RBPT had a value of 39%. Likewise, accuracy was high for all tests (more than 83%) with similar values. ROC AUC values were moderate (63–67%) and close to each other as well.

**Table 5:** Performance evaluation metrics of machine learning^*^ model calculated from prediction scores for the serum tests RBPT, i-ELISA, and real-time PCR.

	**RBPT**	**i-ELISA**	**Real-time PCR**
Precision (PPV)^**^	44%	50%	43%
Recall (NPV)^***^	39%	42%	30%
Accuracy^****^	83%	84%	91%
ROC AUC^*****^	65%	67%	63%

*RBPT, Rose–Bengal agglutination test; PPV, positive predicted value; NPV, negative predicted value; AUC ROC, area under the receiver operating characteristic curve*.

* *Machine learning steps involved supervised learning by decision tree algorithm, classification of animals as positive or negative based on the model of each diagnostic test, and evaluation of the established models by matching the predicted (classified) values as an output from each model and the real results of the diagnostic test*.

** *Precision (random error) is the agreement among repeated analyses of a sample. It is also called positive predicted value. It means the odds that the test method has made a correct prediction when it predicts a positive value*.

*** *Recall (sensitivity or negative predicted value) is how often the test method is making a correct prediction when the actual value is positive*.

**** *Accuracy is nearness of a test value to the actual value. It is the number of correct predictions made as a ratio of all predictions made*.

***** *ROC AUC is the area under the receiver operating curve indicating the ability of a binary classifier to discriminate between positive and negative classes at various diagnostic thresholds. An ROC curve is a graph showing the performance of a test method at all classification thresholds by plotting the true positive rate (on y-axis) vs. the false positive rate (on x-axis)*.

### Relationship Between Risk Factors and Diagnostic Test Results Using Machine Learning

Comparing the results of the three models of diagnostic tests (Figures 2–4) revealed that the lactation attribute was the root node in all tests, indicating that this attribute was significant. The second level after the root node in RBPT and i-ELISA was identical involving both age and abortion risk factors. In real-time PCR test model, abortion was replaced by pregnancy.

## Discussion

Brucellosis is one of the most contagious zoonoses that are still endemic in many countries including Pakistan (1, 16). It is not only an occupational risk to livestock professionals but also a food-borne threat to consumers of animal products (27). Abortions, infertility, and reduction in milk production are adverse effects of brucellosis on livestock production. In developing countries, livestock is the basis of livelihood for about 95% of the rural population (12) providing food, skin, fibers, manure (fertilizer or fuel), and draft power. The identification of *Brucella* spp. in various farm animals and wildlife species highlights their role in disease spread (28–33).

Serological tests are unable to differentiate *Brucella* spp., as antibodies to smooth brucellae lipopolysaccharide (LPS) highly cross-react. Isolation and identification of the etiological agent from biological specimens remain the “gold standard” for epidemiological investigation and determination of antimicrobial resistance, but these methods are time-consuming and hazardous. The detection of *Brucella* DNA by PCR in clinical samples is a preferred tool for conclusive diagnosis of brucellosis (15). Despite the low sensitivity of the real-time PCR resulting from the presence of only trace amounts of DNA in serum, the test achieved the highest accuracy (91%) than the RBPT and i-ELISA (Table 3) known for their sensitivity. *Brucella abortus* DNA was detected in sera from 13 cattle and 18 buffalo cows. This current finding agrees with previous reports indicating the endemicity of *B. abortus* in bovines in Pakistan (17, 21, 32, 34–36). One study also reported *Brucella melitensis* infection in bovines (12). Molecular studies on brucellosis are only limited to certain regions, and scarce literature is available relevant to Pakistan.

Serology remains the practical tool for the diagnosis of brucellosis in bovines. A vast number of serological tests used for screening have been developed starting with a simple qualitative agglutination test and progressing to sophisticated primary binding assays. RBPT is a widely accepted method for screening of anti-*Brucella* antibodies in bovines (1). However, a cross-reaction with antibodies of non-*Brucella* antigens negatively influences specificity (34). False-negative reactions can occur in the RBPT due to prozoning with sera containing very high levels of antibody. In the current study, the RBPT agreed with i-ELSA in the majority of animals (65 positives) with a single cow exclusively positive to the RBPT only, three animals positive to the i-ELISA only, and three animals commonly positive to the i-ELISA and the real-time PCR altogether (Figure 5A). The RBPT false-negative rate, accuracy, and the ROC AUC (39, 83, and 65%) were close to the corresponding values of the i-ELISA (42, 84, and 67%) in that order (Table 3). The RBPT is a suitable rapid screening test for brucellosis, followed by confirmatory testing (13).

The indirect enzyme immunoassays generally have very high sensitivity, but they cannot distinguish *B. abortus* S19 post-vaccinal antibodies. Indirect ELISA proved to be highly sensitive and is the recommended test for brucellosis diagnosis in well-equipped diagnostic facilities (4, 37). Being a quantifier of antibody concentration unrelatedly to its biological activity with a reliable low detection limit (38), the i-ELISA in this study detected the highest number of positives (71, 17.7%) as in Table 3. All in all, the i-ELISA achieved the best diagnostic performance parameters in comparison to the RBPT and real-time PCR (Table 3).

Screening of milk samples using MRT is recommended by the OIE for herd prevalence/freedom of infection (4). However, the diagnostic sensitivity and specificity of MRT are low if compared with milk ELISA (39). The MRT is prone to false reactions caused by abnormal milk such as mastitic milk, colostrums, and milk of the late lactation cycle. Still, in spite of its problems, it is an inexpensive screening test if combined with other tests. Of the 176 lactating bovines tested, the MRT detected 37 (21.02%) positives including a cow exclusively discovered by the MRT (Figure 5B, Table 3).

The prevalences in milk (21.02%) and serum samples (17.7% by i-ELISA) found in this study are slightly higher than in previous reports on bovine brucellosis (0–15%) in Pakistan (20, 21, 40). Contrary to our findings of 16.4 and 17.7% seroprevalences by RBPT and i-ELISA, respectively, few studies reported corresponding seroprevalences of 5.6 and 4.7% by RBPT and i-ELISA, respectively, which were significantly (*p* < 0.05) higher in buffaloes than in cattle (1, 12). Cattle (18.75 and 19.71%) were more often found positive than buffaloes (13.91 and 15.46%) in this study by RBPT and i-ELISA, respectively. These results are in agreement with previous findings (12, 41, 42). Hence, reports with a higher prevalence in buffaloes than in cattle exist (1, 43). The phenomenon toward biological affinity in cattle and buffaloes for brucellosis remains unclear.

Farmers in studied districts (Narowal, Gujranwala, and Gujrat) are used to rear animals in small and medium herds. These areas are irrigated by canals and the rivers of Ravi and Chenab to support the economical production of livestock. This study found a lower disease frequency in regions with a dry environment than in those regions that have access to rivers/irrigation water. This points to the fact that *Brucella* is an environmental contaminant, as illegal disposal of infected materials contributes to the spread of disease as known for the River Nile and its canals in Egypt (44). Instead of culling, infected animals are sold in the markets resulting in further spread of infection. It can be assumed that the incidence of brucellosis in bovines is increasing day by day in Pakistan, which is comparable with the situation in sheep and goats (35).

Statistical association of risk factors with brucellosis test results (RBPT, i-ELISA, and real-time PCR) was studied using chi-squared test. There was no significant relationship recorded (Table 4). The authors, therefore, resorted to an alternative means of investigation, where infectious diseases in general and brucellosis in particular are tricky in the sense that they are unpredictable with so many factors that affect the course of infection and transmission. Machine learning as a branch of artificial intelligence is a very promising tool for monitoring and better understanding of health and disease. One of the uses of machine learning by mathematicians is to assess variables related to infection using a diversity of input data (e.g., location, genus, species, sex and age of animals, herd size, reproductive disease/problem, history of abortion, pregnancy, and lactation).

After building the machine learning model based on decision tree, it was then necessary to assess its performance before its application on the data (Table 5). Each of the performance metrics values of precision, recall, accuracy, and ROC AUC calculated from prediction scores for RBPT, i-ELISA, and real-time PCR were close to each other, indicating that these tests give close results. As to the performance of machine learning model, it achieved an acceptable accuracy over 83%, which is fair enough. Although the PPVs and NPVs were low affecting the model performance as a positive negative classifier, the main objective of building the model was not only to be used as a classifier but also for comparing/arranging the significance of risk factors and their association with seroprevalence.

A higher proportion of anti-*Brucella* antibodies were detected in lactating and pregnant animals. These findings are in agreement with reports from India, Zimbabwe, and Sudan, where the risk of brucellosis increased in pluriparous cows (45–47). Significant high prevalence was also observed in lactating (22.35%) vs. non-lactating (2.46%) cattle from Ethiopia (48). These findings may be caused by the fact that brucellae grow in the gravid uterus of cattle in large numbers due to erythritol affinity to make the gravid uterus a predilection site (49). The ability to catabolize erythritol preferentially over other sugars by bacteria of the genus *Brucella* is associated with the capability to induce abortions in infected ruminants (50). The phenomenon of high prevalence in lactating animals is not well-understood but may be linked to the production stress, which induces clinical infection from latent bacteria residing in the supramammary lymph nodes.

A number of studies in Pakistan documented age, species/breed, the status of animal production/reproduction, history of abortion, and underlying reproductive issues as potential risk factors for brucellosis at the individual or herd level (1, 12, 43, 51–53). These findings are in accordance with the results of the current study. Prevalence was closely associated with age and history of abortion. Animals older than 7 years had higher seroprevalences. These results are in agreement with similar findings from previous reports (45, 54). Younger age may be connected to sexual immaturity and/or passive immunity acquired by maternal antibodies (45).

High seroprevalences were recorded in herds with more than 30 animals in this study. Brucellosis is frequently reported from intensive dairy farms (1). Herd size and geographical location were not found to be associated with brucellosis prevalence in this study, which is in agreement with previous studies in Pakistan (43, 55) but former reports in other parts of the country (1, 12, 53).

A higher seropositivity of brucellosis was observed in animals associated with a history of abortion and underlying reproductive problems (38.5 and 18.7%), respectively. These are the cardinal symptoms of *Brucella* infection, and thus these findings are not surprising. Significant association of brucellosis with the occurrence of abortions in cattle and reproductive problems, i.e., retained fetal membranes, was reported from various regions in Ethiopia (48, 56, 57). Similar results were also reported by other authors (45, 51, 52).

The determination of brucellosis prevalence and associated risk factors is an important determinant to predict the epidemiological status of the disease. Eradication of brucellosis is only possible when positive animals are culled. Consequently, the trade or movement of *Brucella*-positive animals must be prohibited, and a compensation policy must be brought into force to get acceptance of farmers for these measurements. Additionally, biosafety and biosecurity practices must be set in force to decrease the incidence of brucellosis (58).

Not only surveillance and vaccination still remain uncoordinated, but they also constitute a financial limitation in developing countries. Therefore, brucellosis control should take good advantage of the rapid progress in the field of artificial intelligence including machine learning for computational analysis of big epidemiological data aiming for more effective disease management. Machine learning, as one of the necessary components for digitalization of animal health, is expected to open up many possibilities that boost precision livestock industry, health, and well-being, offering new opportunities for future farms.

## Conclusion

Brucellosis remains a lingering infection jeopardizing dairy herds in Pakistan. *Brucella abortus* was identified as a causative agent of bovine brucellosis in upper Punjab, Pakistan. Animals without any sign of illness may remain carriers shedding brucellae into the environment. Machine learning results revealed that the lactation status was the root node in all tests supporting the real findings, while age, abortion, and pregnancy were located in a lower level of significance. To the authors' best knowledge, this is the first time to use machine learning to assess brucellosis risk factors in Pakistan, a model that can be applied for other developing countries in the future. Elimination of positive shedders, development of control strategies, and interactive extension programs for brucellosis are urgently needed in developing countries like Pakistan.

## Data Availability Statement

The raw data supporting the conclusions of this article will be made available by the authors, without undue reservation.

## Ethics Statement

This study was approved by the ethical committee of the College of Veterinary & Animal Sciences, Jhang- University of Veterinary & Animal Sciences, Lahore, Pakistan via approval number CS/481. Oral and written consent was also taken from each farmer before blood and milk sample collection.

## Author Contributions

Data curation was carried out by AK. Formal analysis was performed by AK, AS, and AH. Investigation was done by AK, IK, SE-u-H, UW, MF, SA, and HE-A. Methodology was provided by AK, FM, HN, and HE-A. Supervision was carried out by FM, IK, ME, HN, and HE-A. Writing – original draft was done by AK and HE-A. Writing – review and editing were performed by AK, FM, AH, AS, IK, ME, SE-u-H, UW, MF, SA, HN, and HE-A. All authors contributed to the article and approved the submitted version.

## Conflict of Interest

The authors declare that the research was conducted in the absence of any commercial or financial relationships that could be construed as a potential conflict of interest.

## References

[1] Jamil T Melzer F Saqib M Shahzad A Khan Kasi K Hammad Hussain M. Serological and molecular detection of bovine brucellosis at institutional livestock farms in Punjab, Pakistan. Int J Environ Res Public Health. (2020) 17:1412. 10.3390/ijerph17041412 32098207 PMC7068318

[2] Peng C Zhou H Guan P Wu W Huang DS. An estimate of the incidence and quantitative risk assessment of human brucellosis in mainland China. Transbound Emerg Dis. (2020). 10.1111/tbed.13518 32077219

[3] Kaaboub EA Ouchene N Ouchene-Khelifi NA Khelef D. Serological and histopathological investigation of brucellosis in cattle in Medea region, Northern Algeria. Vet World. (2019) 12:713–8. 10.14202/vetworld.2019.713-718 31327909 PMC6584856

[4] OIE Brucellosis (*Brucella abortus, B. melitensis* and *B. suis*) (infection with *B. abortus, B. melitensis* and *B. suis*) *Manual of Diagnostic Tests and Vaccines for Terrestrial Animals 2019*. Paris: OIE World Health Organization for Animal Health World Organisation for Animal Health (Office International des Épizooties) (2019). p. 355–98.

[5] Hassan H Salami A Nehme N Hakeem RA Hage JE Awada R. Prevalence and prevention of brucellosis in cattle in Lebanon. Vet World. (2020) 13:364–71. 10.14202/vetworld.2020.364-371 32255981 PMC7096292

[6] Liu Z Shen T Wei D Yu Y Huang D Guan P. Analysis of the epidemiological, clinical characteristics, treatment and prognosis of human brucellosis during 2014-2018 in Huludao, China. Infect Drug Resist. (2020) 13:435–45. 10.2147/IDR.S236326 32104015 PMC7023865

[7] Njenga MK Ogolla E Thumbi SM Ngere I Omulo S Muturi M. Comparison of knowledge, attitude, and practices of animal and human brucellosis between nomadic pastoralists and non-pastoralists in Kenya. BMC Public Health. (2020) 20:269. 10.1186/s12889-020-8362-0 32093689 PMC7041083

[8] Khan AU Melzer F El-Soally S Elschner MC Mohamed SA Sayed Ahmed MA. Serological and molecular identification of *Brucella* spp. in pigs from Cairo and Giza governorates, Egypt. Pathogens. (2019) 8:248. 10.3390/pathogens8040248 31756893 PMC6963660

[9] Moreno E Moriyon I The Genus *Brucella*. In: Dworkin M, editor. The Procaryotes: An Evolving Microbiological Resource for the Microbiological Community. 3 New York, NY: Springer (2001). p. 315–457.

[10] Mangtani P Berry I Beauvais W Holt HR Kulashri A Bharti S. The prevalence and risk factors for human *Brucella* species infection in a cross-sectional survey of a rural population in Punjab, India. Trans R Soc Trop Med Hyg. (2020) 114:255–63. 10.1093/trstmh/trz133 32086527

[11] Selim A Attia K Ramadan E Hafez YM Salman A. Seroprevalence and molecular characterization of *Brucella* species in naturally infected cattle and sheep. Prev Vet Med. (2019) 171:104756. 10.1016/j.prevetmed.2019.104756 31520873

[12] Saeed U Ali S Khan TM El-Adawy H Melzer F Khan AU. Seroepidemiology and the molecular detection of animal brucellosis in Punjab, Pakistan. Microorganisms. (2019) 7:449. 10.3390/microorganisms7100449 31614956 PMC6843438

[13] Nielsen K Yu WL. Serological diagnosis of brucellosis. Prilozi. (2010) 31:65–89. 20703184

[14] Mathew C Stokstad M Johansen TB Klevar S Mdegela RH Mwamengele G. First isolation, identification, phenotypic and genotypic characterization of *Brucella abortus* biovar 3 from dairy cattle in Tanzania. BMC Vet Res. (2015) 11:1–9. 10.1186/s12917-015-0476-8 26195218 PMC4508816

[15] Ulu-Kilic A Metan G Alp E. Clinical presentations and diagnosis of brucellosis. Recent Pat Antiinfect Drug Discov. (2013) 8:34–41. 10.2174/1574891X11308010007 22873352

[16] Kirk MD Pires SM Black RE Caipo M Crump JA Devleesschauwer B World Health Organization estimates of the global and regional disease burden of 22 foodborne bacterial, protozoal, and viral diseases, 2010: a data synthesis. PLoS Med. (2015) 12:e1001921 10.1371/journal.pmed.1001921 26633831 PMC4668831

[17] Saddique A Ali S Akhter S Khan I Neubauer H Melzer F. Acute febrile illness caused by *Brucella abortus* infection in humans in Pakistan. Int J Environ Res Public Health. (2019) 16:4071. 10.3390/ijerph16214071 31652718 PMC6862605

[18] Siyal GEA Khalid I Qaisrani A Internal Migration and Urbanization: A Case Study from Semi-arid Regions of Pakistan. Islamabad: SDPI (2018).

[19] Ishfaq S Ahmed V Hassan D Javed A In: Rajan SI Internal Migration and Labour Mobility in Pakistan. Routledge Taylor & Francis (2017).

[20] Ismail M Ahmad I Khan MS Ullah S Malik MI Muhammad K Seroprevalance of *Brucella abortus* in cattle and buffaloes in district Rajanpur, Punjab, Pakistan. Pure Appl Biol. (2018) 7:556–64. 10.19045/bspab.2018.70069

[21] Ali S Neubauer H Melzer F Khan I Akhter S Jamil T Molecular identification of bovine brucellosis causing organisms at selected private farms in Pothohar Plateau, Pakistan. Pakistan J Zool. (2017) 49:1111–4. 10.17582/journal.pjz/2017.49.3.sc2

[22] Soomro A Kamboh AA Rind R Dawani P Sarwar M Abro SH A study on prevalence and risk factors of brucellosis in cattle and buffaloes in district Hyderabad, Pakistan. J Anim Health. (2014) 2:33–7. 10.14737/journal.jahp/2014/2.3.33.37

[23] Virtual Governance System Livestock and Dairy Development Department Avialble online at: http://www.livestockpunjab.gov.pk (accessed February 2020).

[24] Probert WS Schrader KN Khuong NY Bystrom SL Graves MH. Real-time multiplex PCR assay for detection of *Brucella* spp., *B. abortus*, and *B. melitensis*. J Clin Microbiol. (2004) 42:1290–3. 10.1128/JCM.42.3.1290-1293.2004 15004098 PMC356861

[25] Hancock T Jiang T Li M Tromp J Lower bounds on learning decision lists and trees. Inform Comput. (1996) 126:114–22. 10.1006/inco.1996.0040

[26] Fountain-Jones NM Machado G Carver S Packer C Recamonde-Mendoza M Craft ME. How to make more from exposure data? An integrated machine learning pipeline to predict pathogen exposure. J Anim Ecol. (2019) 88:1447–61. 10.1111/1365-2656.13076 31330063

[27] MamaniMMajzoobiMMKeramatFVarmaghaniNMoghimbeigiA. Seroprevalence of brucellosis in butchers, veterinarians and slaughterhouse workers in Hamadan, Western Iran. J Res Health Sci. (2018) 18:e00406.29777092

[28] Godfroid J Al Dahouk S Pappas G Roth F Matope G Muma J. A “One Health” surveillance and control of brucellosis in developing countries: moving away from improvisation. Comp Immunol Microbiol Infect Dis. (2013) 36:8. 10.1016/j.cimid.2012.09.001 23044181

[29] Godfroid J. Brucellosis in livestock and wildlife: zoonotic diseases without pandemic potential in need of innovative one health approaches. Arch Public Health. (2017) 75:1–6. 10.1186/s13690-017-0207-7 28904791 PMC5592711

[30] Musa MT Eisa MZ El Sanousi EM Abdel Wahab MB Perrett L. Brucellosis in camels (*Camelus dromedarius*) in Darfur, Western Sudan. J Comp Pathol. (2008) 138:151–5. 10.1016/j.jcpa.2007.10.005 18346482

[31] Junqueira DG Dorneles EM Goncalves VS Santana JA Almeida VM Nicolino RR. Brucellosis in working equines of cattle farms from Minas Gerais State, Brazil. Prev Vet Med. (2015) 121:380–5. 10.1016/j.prevetmed.2015.06.008 26347382

[32] Jamil T Melzer F Khan I Iqbal M Saqib M Hammad Hussain M. Serological and molecular investigation of *Brucella* species in dogs in Pakistan. Pathogens. (2019) 8:294. 10.3390/pathogens8040294 31847082 PMC6963446

[33] Khan AU Sayour AE Melzer F El-Soally S Elschner MC Shell WS. Seroprevalence and molecular identification of *Brucella* spp. in camels in Egypt. Microorganisms. (2020) 8:1035. 10.3390/microorganisms8071035 32668648 PMC7409340

[34] Abubakar M Mansoor M Arshed M Bovine brucellosis: old and new concepts with Pakistan perspective. Pak Vet J. (2012) 32:147–155.

[35] Ali S Akhter S Neubauer H Melzer F Khan I Ali Q. Serological, cultural, and molecular evidence of *Brucella* infection in small ruminants in Pakistan. J Infect Dev Ctries. (2015) 9:470–5. 10.3855/jidc.5110 25989166

[36] Fatima S Khan I Nasir A Younus M Saqib M Melzer F. Serological, molecular detection and potential risk factors associated with camel brucellosis in Pakistan. Trop Anim Health Prod. (2016) 48:1711–8. 10.1007/s11250-016-1148-9 27677292

[37] Nielsen KH Kelly L Gall D Nicoletti P Kelly W. Improved competitive enzyme immunoassay for the diagnosis of bovine brucellosis. Vet Immunol Immunopathol. (1995) 46:285–91. 10.1016/0165-2427(94)05361-U 7502488

[38] Sanaei Dashti A Karimi A Javad V Shiva F Fallah F Alaei MR. ELISA cut-off point for the diagnosis of human brucellosis; a comparison with serum agglutination test. Iran J Med Sci. (2012) 37:9–14. 23115425 PMC3470292

[39] Godfroid J Nielsen K Saegerman C. Diagnosis of brucellosis in livestock and wildlife. Croat Med J. (2010) 51:296–305. 10.3325/cmj.2010.51.296 20718082 PMC2931434

[40] Gul ST Khan A Ahmad M Rizvi F Shahzad A Hussain I Epidemiology of brucellosis at different livestock farms in the Punjab, Pakistan. Pak Vet J. (2015) 35:309–14.

[41] Abubakar M Javed Arshed M Hussain M Ehtishamul H Ali Q Serological evidence of *Brucella abortus* prevalence in Punjab province, Pakistan–a cross-sectional study. Transbound Emerg Dis. (2010) 57:443–7. 10.1111/j.1865-1682.2010.01171.x 21117286

[42] Farooq U Fatima Z Afzal M Anwar Z Jahangir M Sero-prevalence of brucellosis in bovines at farms under different management conditions. Brit J Dairy Sci. (2011) 2:35–9.

[43] Ali S Akhter S Neubauer H Melzer F Khan I Abatih EN. Seroprevalence and risk factors associated with bovine brucellosis in the Potohar Plateau, Pakistan. BMC Res Notes. (2017) 10:73. 10.1186/s13104-017-2394-2 28129787 PMC5273848

[44] El-Tras WF Tayel AA Eltholth MM Guitian J. *Brucella* infection in fresh water fish: Evidence for natural infection of Nile catfish, Clarias gariepinus, with *Brucella melitensis*. Vet Microbiol. (2010) 141:321–5. 10.1016/j.vetmic.2009.09.017 19880265

[45] Madut NA Muwonge A Nasinyama GW Muma JB Godfroid J Jubara AS. The sero-prevalence of brucellosis in cattle and their herders in Bahr el Ghazal region, South Sudan. PLoS Negl Trop Dis. (2018) 12:e0006456. 10.1371/journal.pntd.0006456 29924843 PMC6010255

[46] Renukaradhya GJ Isloor S Rajasekhar M. Epidemiology, zoonotic aspects, vaccination and control/eradication of brucellosis in India. Vet Microbiol. (2002) 90:183–95. 10.1016/S0378-1135(02)00253-5 12414143

[47] Ndengu M Matope G de Garine-Wichatitsky M Tivapasi M Scacchia M Bonfini B. Seroprevalence of brucellosis in cattle and selected wildlife species at selected livestock/wildlife interface areas of the Gonarezhou National Park, Zimbabwe. Prev Vet Med. (2017) 146:158–65. 10.1016/j.prevetmed.2017.08.004 28992921

[48] Ibrahim N Belihu K Lobago F Bekana M. Sero-prevalence of bovine brucellosis and its risk factors in Jimma zone of Oromia Region, South-western Ethiopia. Trop Anim Health Prod. (2010) 42:35–40. 10.1007/s11250-009-9382-z 19543803

[49] Poester FP Samartino LE Santos RL. Pathogenesis and pathobiology of brucellosis in livestock. Rev Sci Tech. (2013) 32:105–15. 10.20506/rst.32.1.2193 23837369

[50] Yaeger MJ Holler LD Bacterial causes of bovine infertility and abortion. In: Robert S. Youngquist WRT, editors. Current Therapy in Large Animal Theriogenology. St. Louis, MO: Elsevier (2007). p. 389–99. 10.1016/B978-072169323-1.50052-0

[51] Ullah Q Jamil H Lodhi LA Qureshi ZI Ullah S Jamil T Brucellosis is significantly associated with reproductive disorders in dairy cattle of Punjab, Pakistan. Pak J Zool. (2019) 51:1995–7. 10.17582/journal.pjz/2019.51.5.sc10

[52] Arif S Thomson PC Hernandez-Jover M McGill DM Warriach HM Hayat K. Bovine brucellosis in Pakistan; an analysis of engagement with risk factors in smallholder farmer settings. Vet Med Sci. (2019) 5:390–401. 10.1002/vms3.165 30957947 PMC6682800

[53] Arif S Thomson PC Hernandez-Jover M McGill DM Warriach HM Heller J. Knowledge, attitudes and practices (KAP) relating to brucellosis in smallholder dairy farmers in two provinces in Pakistan. PLoS ONE. (2017) 12:e0173365. 10.1371/journal.pone.0173365 28301498 PMC5354373

[54] Liu F Wang D Yang SC Zhu JH Li JM Shi K. Prevalence and risk factors of brucellosis, toxoplasmosis, and neosporosis among Yanbian Yellow cattle in Jilin province, China. Vector Borne Zoonotic Dis. (2019) 19:217–21. 10.1089/vbz.2018.2288 30328792

[55] Shabbir MZ Nazir MM Maqbool A Lateef M Shabbir MA Ahmad A. Seroprevalence of Neospora caninum and *Brucella abortus* in dairy cattle herds with high abortion rates. J Parasitol. (2011) 97:740–2. 10.1645/GE-2734.1 21506829

[56] Tesfaye G Tsegaye W Chanie M Abinet F Seroprevalence and associated risk factors of bovine brucellosis in Addis Ababa dairy farms. Trop Anim Health Prod. (2011) 43:1001–5. 10.1007/s11250-011-9798-0 21331496

[57] Adugna KE Agga GE Zewde G. Seroepidemiological survey of bovine brucellosis in cattle under a traditional production system in western Ethiopia. Rev Sci Tech. (2013) 32:765–73. 10.20506/rst.32.2.2218 24761729

[58] Musallam I Ndour AP Yempabou D Ngong CC Dzousse MF Mouiche-Mouliom MM. Brucellosis in dairy herds: a public health concern in the milk supply chains of West and Central Africa. Acta Trop. (2019) 197:105042. 10.1016/j.actatropica.2019.105042 31152725 PMC6710496

