# Supplementary material for: Seroprevalence and Molecular Identification of Brucella spp. in Bovines in Pakistan—Investigating Association With Risk Factors Using Machine Learning
Source: Front Vet Sci. 2020 Dec 2;7:594498. doi: 10.3389/fvets.2020.594498 (PMC7738322; doi:10.3389/fvets.2020.594498)
Supplement: Supplementary file 1 [file Data_Sheet_1.PDF]

QUESTIONNAIRE FOR BRUCELLA PREVALENCE/ SURVEILLANCE

Sample ID: ____________________ **Date of Collection:** _____________________

Place of collection: _____________________**Name of owner:** ________________________

Contact no. of owner: ____________________**Profession:** ____________________________

Address:

____

Declarations:

I agree to provide the blood sample of my animals for all types of diagnostic research regarding brucellosis or other infectious diseases in my animals.

Signature: ______________

Animal Related Information:

Sample taken from: Cow/cattle

Age: ____________ Weight: ___________

Sex: M / F Lactation no: ___________

Feeding method: ____________ Breeding method: ______________

Age status: heifer/ yearling/ cow calf/

Status of animal: pregnant lactating / dry lactating / dry/ open

Abortion age: ___________ Fetal Viability: yes/ no

Fetal size: _______________ RFM: yes/ no

History of abortion in the herd: yes/ no

If yes, details of abortion: ____

History of stillbirths in the herd: yes/ no

If yes, details of still births: ____

History of infectious diseases in herd: yes/ no

If yes, details of infectious diseases: ____

Vaccination of animals: yes/ no

Vaccines used: ____

Farm to farm distance: ____________________________________

Did you use replacement cows of your own farm: yes/ no

If no, then what is the source of replacement cows?

____

Did you dispose the aborted material in case of any abortion at farm: yes/ no

If yes, then how you dispose of the material?
____

Did you control the movement of birds on farm: yes/ no
Did you have dogs on your farm: yes/ no
If yes, then what is the type of pet animal: stray/ pet/ watch dog

Attendant Related Information:

Profession: veterinarian / abattoir worker / animal attendant
Marital status: married un-married widow (circle one)
Number of children: ____________________________________
Male children:______________________________________
Female children:______________________________________
Any history of abortions: ______________________________
History of joint pain: yes/ no If yes, duration of complaint: ______________
History of muscular pains: yes/ no If yes, duration of complaint: ______________
Complaint of orchitis: yes/ no If yes, duration of complaint: ______________
Complaint of infertility: yes/ no If yes, duration of complaint: ______________

Health related information:

Any major health problem: (circle all that apply) HCV HBV TB Typhoid
Any infection: yes no
X-ray taken in past 3 months: yes no

Exposure to animal:

Years of exposure or keeping of animals: ---
Milk usage: raw/ boiled/ any other ----------------------------
Kind of exposure: Feeding Milking Treatment Other: --------- -----------------

Signature of PI
